# Beyond the Model: Evaluating AI Decision Strategies for Diabetic Retinopathy Screening Under Clinical Constraints

**DOI:** 10.7759/cureus.108630

**Published:** 2026-05-11

**Authors:** Shlok S Jena, Madan M Rayguru

**Affiliations:** 1 Louisville Automation and Robotics Research Institute (LARRI), University of Louisville, Louisville, USA

**Keywords:** artificial intelligence (ai), diabetic retinopathy (dr), early disease detection, machine learning (ml), medical decision-making, retinal imaging

## Abstract

Background

Diabetic retinopathy is one of the leading causes of preventable blindness worldwide, yet it can be stopped through early detection. AI models are increasingly being used as a key enabler to automate this screening, and the results in research settings look very promising. The real challenge is not building more sophisticated models but deploying one that works safely in real clinics. Clinical safety standards require the system to catch nearly every true case of disease, even if that means sending many healthy patients for unnecessary follow-up. This trade-off between keeping patients safe and avoiding a flood of false alarms is the core problem this paper addresses. Given that clinics typically fix a minimum sensitivity target in advance, this study compares decision-making strategies held to the same sensitivity requirement to determine which produces the fewest unnecessary referrals.

Methods

We evaluate five decision strategies under identical conditions on the public EyePACS dataset of 5,270 retinal fundus images, with 1,366 labeled as having diabetic retinopathy. The strategies range from a single AI model making every referral decision independently, to ensemble methods that combine the probability scores of multiple models into one unified output, to a two-tiered method in which all images are first screened by a primary model, and a group of secondary models can overturn a referral when their disagreement is high enough. Each strategy is evaluated under two clinically grounded sensitivity targets, a strict 95% requirement and a more moderate 90% requirement, so the results reflect realistic deployment conditions rather than unconstrained optimal performance.

Results

When the system is required to achieve 95% sensitivity, all strategies produce high false-positive rates, with the best single model reaching only 17.5% specificity. Ensembles offer only marginal improvement at this threshold, while majority voting consistently performs worst across both sensitivity levels. Reducing the sensitivity target from 95% to 90% alone decreases false positives by about 17%. When this lower threshold is combined with an ensemble strategy, unnecessary referrals drop by nearly 25% compared with a single model at 95%. Neither adjustment alone produces this level of improvement; the benefit appears only when both are applied together.

Conclusions

This study shows that two decisions matter most in AI-based diabetic retinopathy screening: the sensitivity target a clinic sets and the decision strategy it pairs with that target. Although the sensitivity target had a greater influence on referral burden, the best outcomes occurred only when both the target and the strategy were carefully chosen. Pairing a 90% sensitivity target with a weighted ensemble reduced unnecessary referrals by nearly 25% compared with a single model at the stricter 95% target, while majority voting produced the highest false-positive burden at both thresholds. These findings suggest that clinically grounded threshold selection is just as important as the decision strategy itself and that seemingly intuitive approaches such as majority voting may underperform when evaluated under the same safety constraints.

## Introduction

Diabetic retinopathy develops without obvious symptoms and can cause lasting damage before a patient notices anything wrong; early and regular screening is essential. The International Diabetes Federation has identified automated screening as a critical public health need, particularly in regions where specialist eye care is limited [1]. Over the past decade, deep learning models for retinal fundus image analysis have shown real promise, with area under the curve (AUC) scores above 0.90 in several large studies [2-4]. A high AUC score alone does not reflect real-world diagnostic quality. A diagnostic tool must reach a certain minimum level of true cases detected for practical purposes in screening programs. Achieving a high true positive rate in the diagnostic tool inevitably leads to hundreds or thousands of false-positive referrals and unnecessary follow-up in a typical mass screening campaign. The research here does not propose a specific model but rather tests a variety of decision-making strategies across an equal set of sensitivities. It compares several decision strategies, each held to the same sensitivity requirement, to find out which produces the fewest unnecessary referrals. Three questions guide the analysis: does the sensitivity threshold chosen, 95% or 90%, matter more than the decision strategy itself; do ensemble methods justify their added complexity; and how does majority voting behave when detection requirements are strict?

Related work

Early research on AI-based DR detection produced impressive results. Gulshan et al. and Ting et al. showed that deep learning models trained on retinal fundus images could match ophthalmologist-level accuracy on large, curated datasets [2,3]. Those findings generated substantial interest and led to the development of increasingly complex architectures, including convolutional networks, vision transformers, and hybrid approaches [5]. The focus throughout was almost entirely on summary accuracy metrics at an unconstrained operating point.

When researchers began testing these systems in actual clinical environments, complications emerged. Abràmoff et al. deployed an autonomous AI screener in primary care offices and found that binary referral decisions, while technically accurate, left clinicians with little control over how the threshold was set or adjusted [4]. Beede et al. documented a different but equally important problem: even a high-performing system can disrupt workflows and create confusion among staff, issues that never appear in benchmark evaluations [6]. On the calibration side, Guo et al. and Ovadia et al. showed that the confidence scores from modern neural networks are often poorly calibrated, meaning a model may assign high probability to predictions that are frequently wrong, which can undermine threshold-based decisions unless explicitly corrected [7,8].

More recent work has begun to take practical deployment constraints seriously. Lim et al. reviewed how camera type and image quality affect AI performance in real programs [9]. Ruamviboonsuk et al. reported on a national screening deployment in Thailand, where sensitivity requirements had to be defined locally and consistently enforced across multiple sites [10]. Wang et al., in a systematic review and meta-analysis of prospective studies, found that real-world performance was consistently lower than what retrospective benchmarks suggested [11].

What is still missing is a direct comparison of decision strategies when all of them are required to meet the same sensitivity target. Ensemble methods have been proposed for improved robustness, and two-stage screening pipelines appear in clinical practice, but neither has been systematically compared against simpler alternatives under a shared safety constraint. Without that comparison, there is no clear evidence for whether the added complexity of an ensemble or a multi-stage pipeline actually pays off operationally.

Study objectives

The primary objective of this study is to determine, under a fixed clinical sensitivity constraint, which decision-making strategy produces the fewest unnecessary referrals in this retrospective EyePACS analysis of retinal fundus images for diabetic retinopathy screening. As secondary questions, we look at (1) whether ensemble methods improve specificity enough to be worth the added complexity, and (2) how majority voting performs when sensitivity requirements are strict. The primary objective shapes the main comparison in the Results, while the two secondary questions help explain why some strategies work better than others.

## Materials and methods

System architecture

The pipeline presented here rests on a basic assumption: safety and efficiency are not to be managed concurrently, but rather independently. This setup aligns with the structure of several clinical review workflows: a sensitive primary screener to flag possible cases conservatively, followed by a second-tier filter to discard a portion of those cases that might not warrant referral. The pipeline thus has two configurations, corresponding roughly to the extremes of the clinical spectrum in which a screening program might operate. The high-safety setting requires at least 95% sensitivity and is intended for scenarios where the consequences of failing to detect a case outweigh those of unnecessary referral. The balanced setting sets a target sensitivity of 90% and may serve as a starting point for settings that are prepared to risk a small increase in missed cases to achieve a reduction in unnecessary referrals. In either case, decision thresholds are chosen on the validation or calibration split to reach the target sensitivity while maximizing specificity.

To generate the scores used for decision-making, each retinal image is fed into three separately trained deep learning architectures: a ResNet50, a ViT-B/16, and a Supervised Convolutional Encoder. Each of these models outputs a five-class severity prediction, with probabilities that the images fall into the respective categories. These probabilities are recalibrated using temperature scaling, which adjusts overconfident probabilities without altering model predictions [7,8]. A single referral probability was determined from the recalibrated probabilities for each of the three models, enabling thresholds to be defined across models.

**Figure 1:**
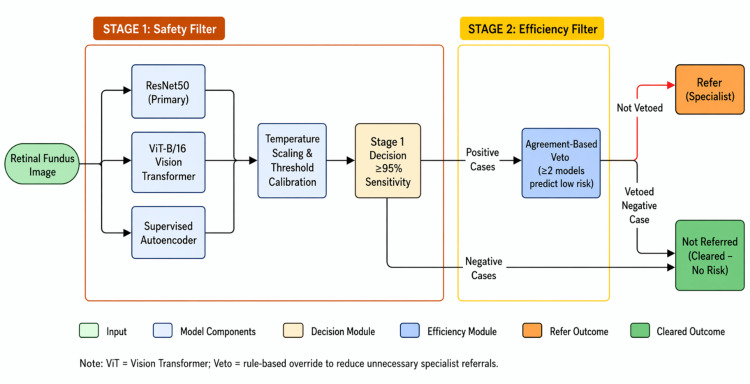
End-to-end screening pipeline showing preprocessing, model inference, temperature calibration, and the two-stage agreement-based veto used for deployment decisions

Stage 1 is always active. Models are calibrated to the required sensitivity level, screen every image, and make an initial referral decision. Images flagged as positive move to Stage 2; images flagged as negative are cleared without further review. Stage 2 applies an agreement-based veto. A positive flag from Stage 1 is overturned only if at least two of the three models independently score the image below a tuned low-risk threshold. The standard for overturning is deliberately high: disagreement from a single model is not enough. If no veto threshold can be set without pushing sensitivity below the required minimum, Stage 2 does not activate and Stage 1 decisions pass through unchanged. Patient safety is not traded for efficiency (Figure 1).

Dataset and preprocessing

Images came from the EyePACS Diabetic Retinopathy Detection challenge via Kaggle [12], which provides retinal fundus images labeled across five DR severity grades. For this study, grades 1 through 4 were treated as referable disease and grade 0 as no disease, creating a binary classification task. Including mild cases in the positive class makes the problem harder, but the comparisons between strategies hold regardless of exactly where the referral line is drawn.

This study involved only secondary analysis of de-identified, publicly available data obtained from the EyePACS dataset. No patient contact was involved, and no identifiable information was accessed. In accordance with applicable regulations, formal institutional review board review was not required for this type of study.

Each image was preprocessed the same way: automatic cropping of dark borders, histogram equalization to improve contrast, and resizing to 224 × 224 pixels. A patient-level three-way split was used to prevent data leakage. The processed dataset of 35,126 images from 17,563 patients was divided into 23,884 training images from 11,942 patients, 5,972 calibration images from 2,986 patients, and 5,270 held-out test images from 2,635 patients. The split was generated using a two-stage GroupShuffleSplit procedure with a fixed random seed of 1337, holding out 15% of patients for testing and then 20% of the remaining patients for calibration. Because EyePACS contains images of both eyes from the same patients, splitting by image rather than by patient would allow one person to appear in both training and testing, inflating performance estimates. Under the patient-level split, the training set was used only for learning model weights, the calibration set was used for threshold selection, ensemble fitting, and veto tuning, and the held-out test set was reserved entirely for final evaluation. The test set was never accessed during any tuning step.

The full processed dataset consisted of 35,126 images from 17,563 patients, and we used a strict patient-level three-way split with no patient overlap between groups. This gave us 23,884 images from 11,942 patients for training, 5,972 images from 2,986 patients for calibration, and 5,270 images from 2,635 patients for the held-out test set. All of the final model comparisons reported in the paper were run on this test set, which contains 1,366 diabetic retinopathy cases. The training set was used only to learn model weights. The calibration set handled threshold selection, ensemble fitting, temperature scaling, and veto tuning. The test set was kept aside entirely until the very end. To make the split itself reproducible, we used a two-stage GroupShuffleSplit, holding out 15% of patients for testing and then taking 20% of the remaining patients for calibration, all with a fixed random seed of 1337.

Data augmentation

Several augmentation techniques were applied during training to help the models generalize. Each image was randomly cropped at a scale between 0.85 and 1.0, flipped horizontally with 50% probability, and rotated by up to 10 degrees in either direction. Color jitter varied brightness, contrast, and saturation, and mild perspective distortion was added. All transformations were constrained to preserve the diagnostic features of retinal images. Pixel values were normalized using standard ImageNet statistics to match the expected input range of the pretrained backbones.

Model architectures

Three architectures were used to represent different feature extraction approaches. ResNet50 is a convolutional network that builds representations through stacked residual blocks, making it effective at detecting local image patterns. ViT-B/16, introduced by Dosovitskiy et al. [5], splits each image into 16 × 16 pixel patches and models relationships between them using self-attention, giving it a more global perspective than convolutional networks. The supervised encoder, based on ResNet18, is a smaller and computationally lighter alternative. All three were pretrained on ImageNet and then fine-tuned for five-class DR severity classification. Training used AdamW optimization with cosine annealing for the learning rate, weighted cross-entropy loss, class-balanced sampling, weight decay of 0.0001, and mixed precision when the hardware supported it. Early stopping was applied with a patience of four epochs. The batch size was 16 for training and 64 for calibration and test inference. Per-model maximum epochs and learning rates differed slightly: ResNet50 was trained for up to 14 epochs at a learning rate of 0.0002, ViT-B/16 for up to 14 epochs at 0.00005, and the supervised encoder for up to 12 epochs at 0.0002. After training, class predictions from each model were converted into continuous probability scores for use in the decision strategies.

All three models were trained on 224 × 224 images with AdamW, cosine annealing for the learning rate, weighted cross-entropy loss, class-balanced sampling, weight decay of 0.0001, and mixed precision where the hardware supported it. We used early stopping with a patience of four epochs throughout. Training ran with a batch size of 16, while calibration and test inference used a batch size of 64. The per-model settings differed slightly: ResNet50 was trained for up to 14 epochs at a learning rate of 0.0002, ViT-B/16 for up to 14 epochs at 0.00005 (transformers tend to need a smaller learning rate for stable fine-tuning), and the supervised encoder for up to 12 epochs at 0.0002.

Probability calibration and threshold selection

Neural networks are often overconfident, assigning high probabilities to predictions that turn out to be wrong more often than the score suggests [7,8]. Temperature scaling corrects this by learning a single scaling parameter for each model using the calibration set. The adjustment changes how confident the outputs sound without altering the order of the predictions.

Each model’s temperature was fit separately on the calibration set using LBFGS with a learning rate of 0.25, a maximum of 50 iterations, and the temperature constrained between 0.5 and 10.0. Values above 1.0 sharpen overconfident outputs, while values below 1.0 sharpen underconfident outputs; either direction is handled by the same fitting procedure. The fitted values were T = 0.83 for ResNet50, T = 0.69 for ViT-B/16, and T = 1.90 for the supervised encoder.

Once probabilities were calibrated, decision thresholds were selected on the calibration set. For each model and strategy, the threshold was set to the lowest value that met the required sensitivity, with specificity maximized subject to that constraint. Those thresholds were then applied to the held-out test set without any further adjustment.

For temperature scaling, we fit a separate scalar for each model on the calibration set using LBFGS with a learning rate of 0.25, a maximum of 50 iterations, and the temperature constrained between 0.5 and 10.0. It is worth noting that temperature values above 1.0 sharpen outputs that were overconfident, while values below 1.0 sharpen outputs that were underconfident; both directions are handled by the same fitting procedure. In our case, the fitted values came out to T = 0.83 for ResNet50, T = 0.69 for ViT-B/16, and T = 1.90 for the supervised encoder. For the Stage 2 veto threshold in the two-stage pipeline, we ran a grid search on the calibration set across 66 values from 0.05 to 0.70, with the rule that at least two of the three models had to fall below the veto threshold before a Stage 1 referral could be reversed.

Decision strategies

Five strategies were evaluated using the calibrated probability outputs of the three models. In single-model thresholding, each model made decisions independently using its own calibrated threshold. No information from the other models was used.

Majority consensus converted each model’s calibrated threshold into a binary vote. A case was flagged if at least two of the three models voted for referral. Because this produces binary outputs rather than continuous scores, AUC cannot be computed for it.

Weighted probability averaging combines each model’s referral probability into a single score by weighting each model according to its validation-set AUC. A single threshold was then applied to the combined score.

Meta-learned stacking trained a logistic regression meta-classifier on the calibration set using K-fold cross-validation to generate unbiased out-of-fold predictions, using the three models’ probability scores as input features. On the held-out test set, the stacking classifier achieved an AUC of 0.769.

The two-stage pipeline follows the architecture described in the previous section: ResNet50 as the Stage 1 screener, with the multi-model veto applied in Stage 2. The veto threshold was identified by grid search on the calibration set across 66 values from 0.05 to 0.70, with the rule that at least two of the three models had to score below the veto threshold before a Stage 1 referral could be reversed.

Evaluation metrics and statistical analysis

Performance on the test set was measured using sensitivity, specificity, AUC, total referral counts, and false positive counts. Referral burden per 1,000 screenings was also computed. AUC was calculated only for strategies that produce continuous probability scores; majority consensus does not, so no AUC is reported for it. CIs for sensitivity and specificity used the Wilson score method, and false positive and referral counts used the Poisson approximation. All calibration and fitting decisions were made using only the calibration set.

Code availability

The full analysis pipeline, including preprocessing, model training, probability calibration, threshold selection, ensemble evaluation, and two-stage veto tuning, was implemented in Python. The code, along with the specific library versions used, will be made publicly available through a GitHub repository upon acceptance. The EyePACS dataset is publicly available through the Kaggle Diabetic Retinopathy Detection challenge.

## Results

Base model performance

**Figure 2:**
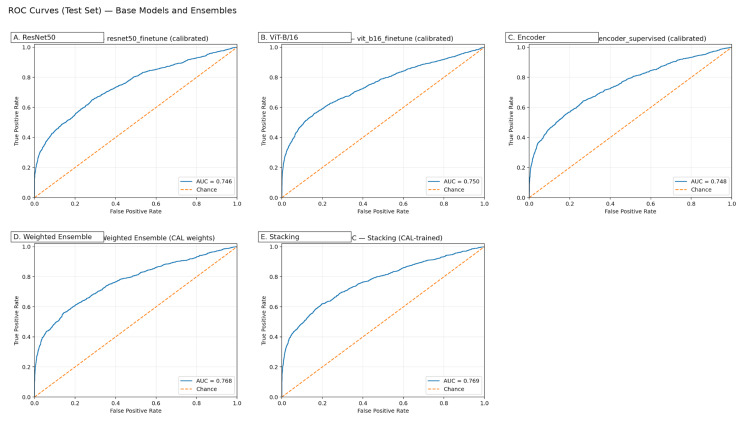
ROC curves on the held-out test set for the three base models and the two ensemble strategies (weighted ensemble and stacking) ROC, receiver operating characteristic

**Figure 3:**
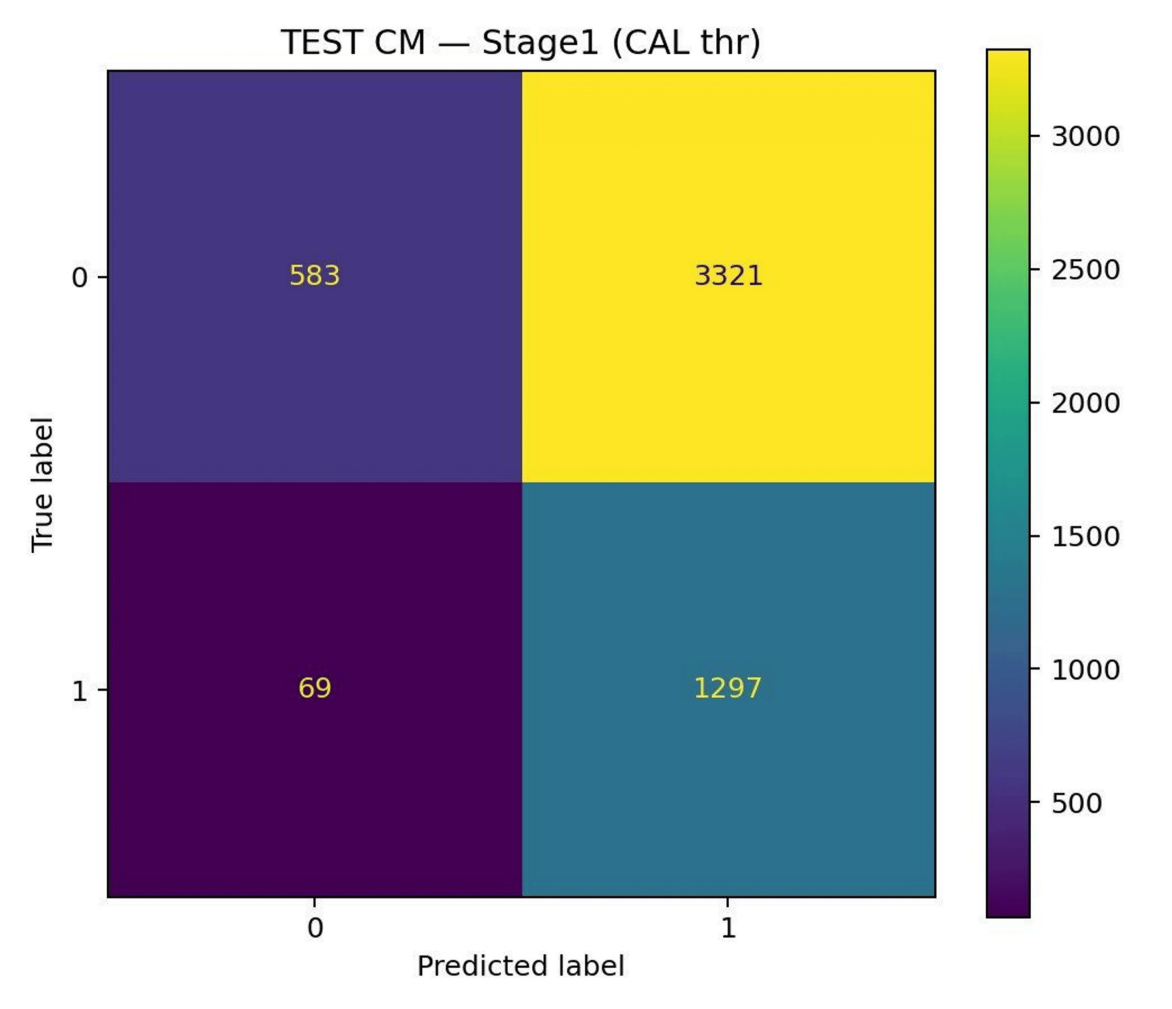
Confusion matrix for ResNet50 at the calibrated 95% sensitivity operating point

The three base models were evaluated on the held-out test set of 5,270 images, which included 1,366 DR cases, a prevalence rate of 25.9%. AUC scores were closely grouped: ViT-B/16 reached 0.750, the supervised encoder 0.748, and ResNet50 0.746 (Figure 2). Despite having the lowest AUC, ResNet50 serves as the main reference point throughout the analysis. It acts as the Stage 1 screener in the two-stage pipeline, and it represents the kind of widely deployed convolutional baseline that most programs would start from. At the 95% sensitivity threshold, ResNet50 produced 1,297 true positives, 3,321 false positives, 69 false negatives, and 583 true negatives (Figure 3).

High-safety operating point (95% sensitivity target)

At 95% sensitivity, the three single models differed more in specificity than their similar AUC scores suggested. The supervised encoder led at 17.5% (95% CI: 16.4-18.7%) with 3,220 false positives. ResNet50 followed at 14.9% (95% CI: 13.8-16.1%) with 3,321 false positives, and ViT-B/16 reached 15.2% (95% CI: 14.1-16.3%) with 3,312. Importantly, ViT-B/16 had the highest overall AUC but not the highest specificity at this operating point. That gap shows why AUC alone is a poor predictor of which model will produce fewer false positives when sensitivity is fixed.

Both ensemble methods, weighted averaging and stacking, reached 16.2% specificity and generated around 3,272 to 3,273 false positives. That is a modest improvement over ResNet50, roughly 50 fewer false positives, but still well below the supervised encoder. Adding model complexity at this sensitivity level provides a small benefit, not a decisive one.

Majority consensus went in the opposite direction. Sensitivity climbed to 95.9% (95% CI: 94.7-96.8%), but specificity dropped to 12.6% (95% CI: 11.6-13.7%), producing 3,413 false positives, the highest total of any strategy. Requiring agreement from only two of three models captured more borderline true positives but swept in far more false positives at the same time. At strict sensitivity levels, majority voting makes the referral problem worse, not better.

The two-stage pipeline achieved 15.3% (95% CI: 14.2-16.4%) specificity with 3,308 false positives. The veto mechanism had very little room to operate at this threshold, so the improvement over ResNet50 alone was marginal.

**Figure 4:**
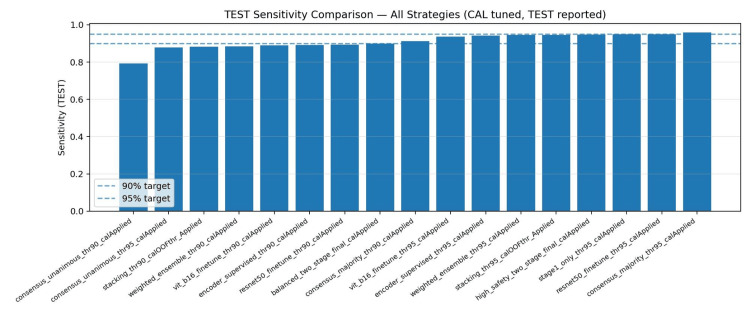
Sensitivity across base models, ensemble strategies, and two-stage pipelines at the 95% and 90% operating points on the held-out test set

Table 1 summarizes results for all strategies at the 95% sensitivity target, and Figure 4 shows achieved sensitivity across all strategies at both the 95% and 90% operating points.

**Table 1:** Decision strategy comparison at the 95% sensitivity target Values are point estimates with 95% CIs calculated using the Wilson score method. Thresholds were selected on the calibration set; actual test-set sensitivity may differ slightly. AUC is not reported for majority consensus, which produces binary outputs. AUC, area under the curve

Method	Sensitivity % (95% CI)	Specificity % (95% CI)	AUC	Referrals	False positives (95% CI)	Referral rate (%)
ResNet50	94.9 (93.7-96.0)	14.9 (13.8-16.1)	0.746	4,618	3,321 (3,208-3,433)	87.6
ViT-B/16	93.7 (92.3-94.9)	15.2 (14.1-16.3)	0.75	4,592	3,312 (3,199-3,424)	87.1
Supervised encoder	94.1 (92.8-95.3)	17.5 (16.4-18.7)	0.748	4,506	3,220 (3,108-3,331)	85.5
Majority consensus	95.9 (94.7-96.8)	12.6 (11.6-13.7)	N/A	4,723	3,413 (3,298-3,527)	89.6
Weighted ensemble	94.7 (93.3-95.7)	16.2 (15.1-17.4)	0.768	4,565	3,272 (3,159-3,384)	86.6
Stacking (CV)	94.7 (93.3-95.7)	16.2 (15.0-17.4)	0.769	4,566	3,273 (3,160-3,385)	86.6
Two-stage (95%)	94.7 (93.4-95.8)	15.3 (14.2-16.4)	0.768	4,602	3,308 (3,195-3,420)	87.3

Balanced operating point (90% sensitivity target)

Dropping the sensitivity target to 90% opened up much larger specificity gains across every strategy. Achieved test-set sensitivities ranged from 88.1% to 91.3%, which is a normal variation from applying calibration-set thresholds to a different data split.

ResNet50’s specificity jumped from 14.9% to 29.2% (95% CI: 27.8-30.7%), cutting false positives from 3,321 to 2,763, a 16.8% reduction. The supervised encoder again led among single models at 30.9% (95% CI: 29.5-32.4%) with 2,696 false positives.

At the 90% target, ensemble methods produced a clear specificity advantage over the single models. The weighted ensemble reached 35.1% (95% CI: 33.6-36.6%) specificity with 2,534 false positives compared to the ResNet50 baseline at the 95% target; those numbers represent 787 fewer referrals across the test set, a 23.7% reduction.

Majority consensus improved at this target, reaching 26.8% (95% CI: 25.4-28.2%) specificity and 2,858 false positives. However, it still produced more referrals than the weighted ensemble, the stacking classifier, and the supervised encoder operating alone.

Table 2 summarizes results for all strategies at the 90% sensitivity target.

**Table 2:** Decision strategy comparison at the 90% sensitivity target Values are point estimates with 95% CIs calculated using the Wilson score method. AUC is not reported for majority consensus. AUC, area under the curve

Method	Sensitivity % (95% CI)	Specificity % (95% CI)	AUC	Referrals	False positives (95% CI)	Referral rate (%)
ResNet50	89.3 (87.6-90.8)	29.2 (27.8-30.7)	0.746	3,983	2,763 (2,659-2,866)	75.6
ViT-B/16	88.9 (87.2-90.5)	29.1 (27.7-30.5)	0.75	3,983	2,768 (2,664-2,871)	75.6
Supervised encoder	89.2 (87.5-90.8)	30.9 (29.5-32.4)	0.748	3,915	2,696 (2,594-2,797)	74.3
Majority consensus	91.3 (89.7-92.7)	26.8 (25.4-28.2)	N/A	4,105	2,858 (2,753-2,962)	77.9
Weighted ensemble	88.4 (86.6-90.0)	35.1 (33.6-36.6)	0.768	3,742	2,534 (2,435-2,632)	71
Stacking (CV)	88.1 (86.3-89.7)	34.9 (33.4-36.4)	0.769	3,747	2,543 (2,444-2,641)	71.1
Two-stage (90%)	89.9 (88.2-91.4)	30.8 (29.4-32.3)	0.768	3,928	2,700 (2,598-2,801)	74.5

Referral burden and cost considerations

Assuming an illustrative saving of $250 per unnecessary referral avoided, switching from the ResNet50 baseline at 95% sensitivity to the weighted ensemble at 90% sensitivity saves approximately $37,250 per 1,000 screenings. This figure will vary across healthcare systems, but institutions can substitute their own local cost data into the same calculation to produce a relevant estimate.

Summary of trade-offs

One pattern ran consistently through every comparison: the sensitivity target drove referral burden more than any choice of model or ensemble strategy. At 95% sensitivity, the best single model outperformed the best ensemble on specificity. At 90%, ensembles pulled ahead, but the biggest jump in efficiency still came from changing the threshold, not the strategy.

Majority voting stood out as the approach that most consistently underdelivered on what its name implies. Its design lowers the effective detection bar below any individual model’s threshold, which at high sensitivity levels means more true positives and a lot more false positives. It was the highest-referral strategy in both scenarios.

The two-stage pipeline performed predictably: limited gains at 95%, where the veto had little room to activate, and moderate gains at 90%. Its value is not mainly in raw specificity numbers but in its structural transparency, which makes it easier to audit and explain to clinical or regulatory audiences.

## Discussion

Practical implications for deployment

At 95% sensitivity, a single well-calibrated model is the most practical choice. Prior clinical deployments have shown that even a straightforward autonomous screener can meet sensitivity requirements in primary care settings without the overhead of ensemble infrastructure [4]. At this sensitivity, the supervised encoder achieved the highest specificity out of all the ensembles, while the cost and effort of implementing and maintaining multiple models could hardly be justified for the incremental benefit they add, a concern echoed in evaluations of real-world AI screening complexity [6].

If the referral capacity is more limited and a sensitivity target of 90% can be justified clinically, then within this dataset and at the AUC range of the base models tested here, ensemble methods do start to look more attractive, with both weighted averaging and stacking providing a clear improvement over a single model. This pattern aligns with findings from large-scale prospective deployments where balancing sensitivity against referral burden was a central operational concern [10,11]. The two-stage pipeline is well suited for cases where we want to understand or audit individual referrals, even if its specificity advantage is only modest, particularly because its structured separation of screening and confirmation stages is easier to explain to clinical teams and regulators [6].

One specific note: Majority voting, for all its intuitive appeal and transparency, results in the largest number of unnecessary referrals at both sensitivity levels. Its interpretability and transparency may nonetheless be useful for governance and regulatory processes where explainability of individual decisions is required [4,6]. Thus, if a clinic wants to use majority voting, it should first test it on real data to ensure the sensitivity can be maintained. In all, the optimal referral strategy is dependent on the specific requirements, capabilities, and context of each particular setting, from the required sensitivity level to the available referral capacity and the complexity that the organization can sustain, a finding consistent with the heterogeneity of outcomes observed across real-world screening programs [9,10].

Deployment risks

The model accuracy, with an AUC score of about 0.746-0.769, is acceptable but does not reflect the complete picture when a system is used clinically. These AUC values are lower than those reported in landmark benchmark studies [2,3] and reflect the well-documented gap between retrospective performance and prospective deployment [11]. Real-world AI screening systems have consistently demonstrated reduced performance relative to benchmark evaluations [4,6]. In a system where we are required to catch 95% of all disease cases, we will inevitably also send many healthy patients to the doctor, without the model making a mistake. Simply, when we require 95% detection and approximately one in four people has the disease, this will be the outcome. This is useful information when the clinic sets up expectations, and it is not a failure of the model.

Majority voting also has its own risks that are easy to overlook. Since a referral decision only needs two of the three models to agree on a referral, it is not a true reflection of the individual model’s sensitivity. In the higher sensitivity settings, the model’s sensitivity can be high, but the referral will have more false positives, even though there is only one referral. If only the sensitivity is tracked, the higher false positives will stay unknown, but if the specificity is also tracked, the true referrals will be noticed.

Decision framework for institutions

Different healthcare settings don’t all operate the same way, so the screening strategy should match the resources and priorities of the institution. High-prevalence or underserved settings in particular may justify aggressive sensitivity targets to minimize missed cases, as has been documented in large-scale national screening programs [1,10]. If an institution is a high-risk area where a false negative could be harmful, and if there are enough resources to receive the referrals, then a screening setting with 95% sensitivity is appropriate. In such settings, the results from this study suggest that a single calibrated model may be appropriate, though external validation is needed before this should be treated as a general deployment recommendation, even though the number of referrals increases [4].

If referral resources are not abundant and institutions want to optimize specificity and minimize referrals, then lowering the threshold to 90% and using an ensemble of models is a reasonable choice. The ensemble will not decrease in performance as long as its threshold is calibrated accordingly. In resource-constrained environments, simplicity becomes more important, as noted in national screening deployments where local calibration of sensitivity thresholds was critical [9,10]. Within this study, a single model with a properly calibrated threshold offered a practical balance of simplicity and performance. Whether this balance holds in other settings will need confirmation through prospective evaluation.

Limitations

This study was evaluated on one dataset, which is a narrow window. Results may differ across other patient populations, imaging systems, or clinical environments, a limitation well established in the AI screening literature, where prospective real-world performance has consistently fallen below retrospective benchmarks [9,11]. External validation in a live screening program is needed before these findings can be treated as definitive.

The base-model AUC range of 0.746-0.769 is lower than that reported in landmark benchmark studies, which has two implications for how the results should be interpreted. The absolute specificity values reported at each sensitivity target are partly a function of this discriminative ceiling and would likely be higher with stronger base models. The relative ranking of strategies, however, is internally controlled within this study because all strategies were evaluated using the same model outputs, calibration procedure, and held-out test set. At the same time, the specific patterns observed here, including the marginal-versus-substantive value of ensembles at the two operating points, should not be assumed to transfer identically to higher-performing systems, where the gap between strategies may compress as base-model AUC improves. Validation in settings with stronger base models is needed before treating these patterns as broadly generalizable.

The $250 referral cost figure is illustrative rather than evidence-based and is presented as a hypothetical example only. Actual referral costs vary substantially across healthcare systems, and any real institutional decision would require locally appropriate cost data. The difference between referral strategies, however, does not depend on the exact dollar figure used.

The stacking classifier performed well with an AUC of 0.769, but the calibration set was small. With more data, the gap between stacking and weighted averaging may look different.

The comparative claims in this study are based on the magnitude of observed differences between strategies and on the reported CIs (Wilson intervals for sensitivity and specificity, Poisson intervals for false positive counts), rather than on formal pairwise hypothesis tests. Because all strategies were evaluated on the same test set with the same input data and shared calibration, they are not independent comparisons, and standard between-group tests would not directly apply. Paired tests such as McNemar’s may be more appropriate for some binary outcome comparisons, but applying these across multiple strategies and operating points with appropriate multiple-comparison handling is beyond the scope of the present analysis. The comparative findings should therefore be interpreted as descriptive within-study comparisons supported by effect size and CIs, rather than as formal hypothesis-tested superiority claims.

This study does not tackle implementation issues. Approval of regulations, integration into the system, staff training, and patient education are also important topics that are beyond the scope of this study.

## Conclusions

A high AUC is not enough. The findings from this retrospective analysis of the EyePACS dataset suggest that the real measure of how well an AI screening system may work in practice comes from a combination of decision strategy and sensitivity target, and of those two, the sensitivity target carries more weight. These conclusions describe the relative behavior of decision strategies on the EyePACS dataset under specific operating conditions and at the AUC range achieved by the base models in this study. Whether the same patterns hold across other populations, imaging hardware, and clinical environments remains an open question, and external validation in prospective settings is needed before any of these patterns can be treated as broadly generalizable. Within this dataset, at 95% sensitivity, the supervised encoder outperformed every ensemble with 17.5% specificity, indicating that a single well-calibrated model may match or exceed more complex approaches under strict constraints. At 90% sensitivity, weighted averaging and stacking pulled ahead, together reducing false positives by 787 compared to the ResNet50 baseline at the stricter target. Majority voting was the weakest-performing strategy at both thresholds tested, producing the highest false positive counts in each scenario evaluated on this dataset. The two-stage pipeline did not lead to specificity within this dataset, but its structured design, where a primary screener and a secondary veto operate independently, may make individual decisions easier to audit and explain than a single score produced by an ensemble. Whether this structural transparency translates into meaningful advantages for clinical governance or regulatory review is a question this study could not address and warrants dedicated prospective evaluation.

Testing these strategies on other diverse datasets from different clinical settings, camera systems, and patient populations would confirm whether the patterns found here hold broadly or are specific to the EyePACS dataset. A prospective study that deploys one of these strategies in a live screening program and tracks real outcomes over time will provide evidence that a static test set simply cannot. On the financial side, a more detailed cost analysis that accounts for downstream factors such as specialist wait times, secondary procedures, and quality-adjusted life-years would give institutions sharper tools for making the sensitivity trade-off with confidence. The same comparison framework could also be applied to other medical imaging tasks, such as chest X-ray review or skin lesion triage, to test whether the dominance of threshold choice over strategy choice extends beyond diabetic retinopathy.
